# Endobronchial Tuberculosis: A Rare Presentation

**DOI:** 10.7759/cureus.8033

**Published:** 2020-05-08

**Authors:** Nadeem M Kassam, Omar M Aziz, Samina Somji, Grace Shayo, Salim Surani

**Affiliations:** 1 Internal Medicine, Aga Khan University Medical College, Dar es Salaam, TZA; 2 Internal Medicine, Aga Khan Hospital, Dar es Salaam, TZA; 3 Internal Medicine, Muhim Bili University of Health and Allied Sciences, Dar es Salaam, TZA; 4 Internal Medicine, Corpus Christi Medical Center, Corpus Christi, USA; 5 Internal Medicine, University of North Texas, Dallas, USA

**Keywords:** endobronchial tuberculosis, bronchoscopy

## Abstract

Endobronchial tuberculosis (EBTB) is an infection of the tracheobronchial tree by Mycobacterium tuberculosis. It is common among young females. Patient can present with fever, cough, wheeze, with or without any constitutional symptoms. It presents as a diagnostic dilemma, as patient sputum smear can be false negative. CT scan may or may not show any abnormality, or any endobronchial lesion. Bronchoscopy with bronchoalveolar lavage and biopsy offers the diagnostic choice. We hereby report a case of a young immunocompetent Asian female who was found to have endobronchial pathology, leading to diagnosis and timely therapy.

## Introduction

Endobronchial tuberculosis (EBTB) is the *Mycobacterium tuberculosis* infection of the tracheobronchial tree. Bronchoalveolar lavage and biopsy helps in the diagnosis [1-2]. EBTB occurs in about 10%-40% of patients with active tuberculosis, however, its pathogenesis remains unclear. It is hypothesized to involve spread from parenchymal lesions or bronchial invasion from mediastinal tuberculous lymphadenitis [3]. More than half the cases of EBTB occur in patients aged less than 35 years old [4-5] and it can certainly be mistaken amongst other diseases as the presentation and clinical findings are nonspecific. The disease progresses as a common complication of active tuberculosis and causes some degree of bronchial stenosis in more than 90% of the patients [6]. It is, however, an extremely infectious disease that poses a diagnostic challenge because of its nonspecific nature and varying clinical findings [5]. Bronchial stenosis caused by the concentric scarring can be fatal in patients with EBTB. The standard treatment is with anti-tubercular medications and the prevention of airway stenosis. Diagnostic modalities include X-ray chest, CT scan, bronchoscopy, but the sputum examination remains the initial diagnostic test. Once that is negative, further investigation can be undertaken with imaging study and diagnostic bronchoscopy with bronchoalveolar lavage and biopsy. We hereby report a case of EBTB in an immunocompetent young Asian female. 

## Case presentation

A 17-year-old immunocompetent Asian female with no comorbid conditions presented to the ED with complaints of acute onset of hemoptysis (50-75 mL). She reported no history of fever, chills, or rigors. The patient denied shortness of breath, chest tightness, unintentional weight loss, or night sweats. She had no contact with anyone who was known or suspected to have tuberculosis.

On clinical examination she was thin, well-oriented, and lying comfortably on the examination bed. General physical examination revealed mild pallor with no signs of cyanosis, clubbing or peripheral lymphadenopathy. Her respiratory rate was 18 breaths/min. On respiratory system examination, the patient had decreased tactile vocal fremitus at middle and lower left lobes. On auscultation, there were bronchial breath sounds with pronounced crepitations on the left from mid to lower zones. The rest of the physical examination was within normal limits.

**Figure 1:**
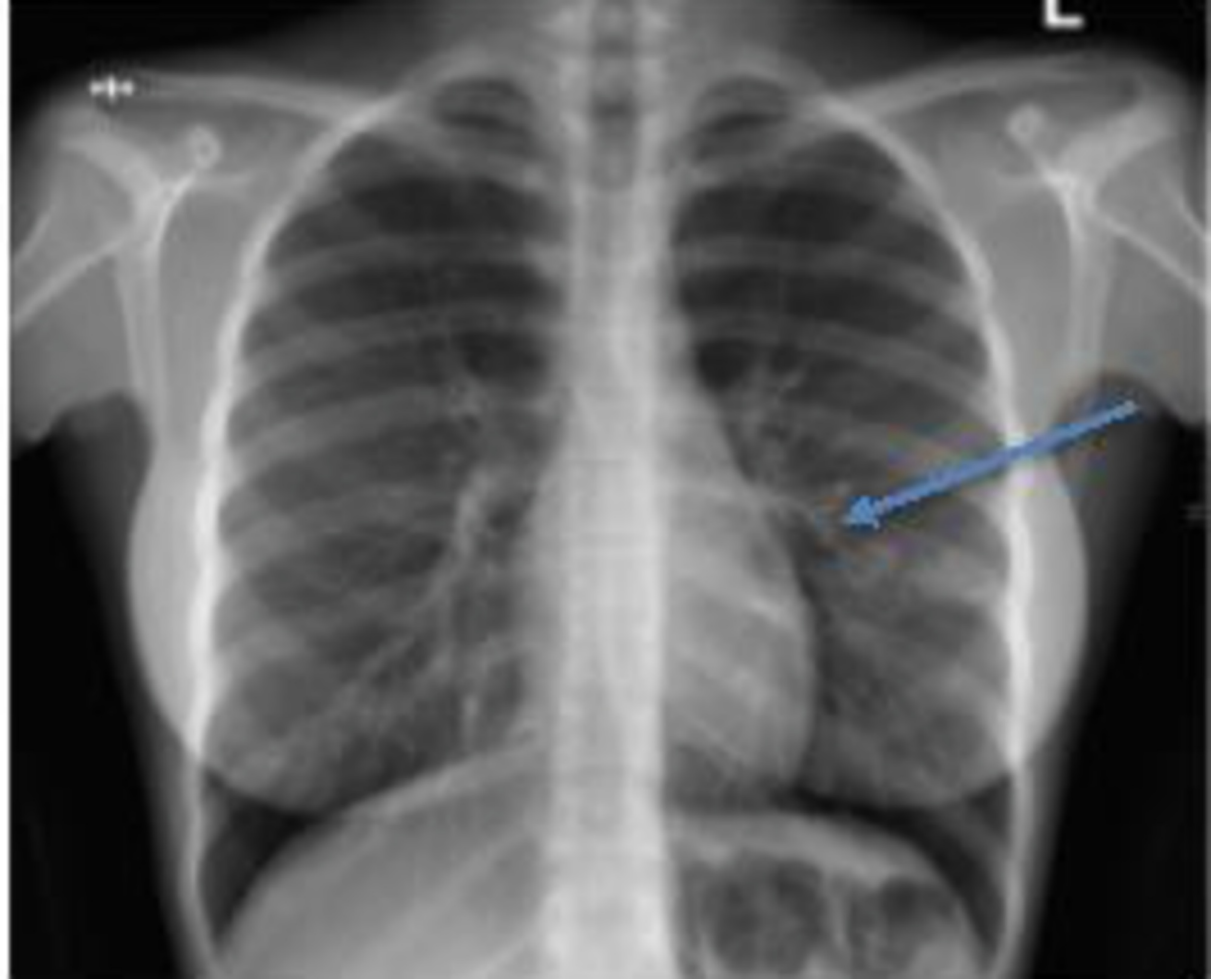
Chest X-ray showing nonhomogeneous cystic and nodular infiltrates at the left mid zone.

Her laboratory investigations revealed normal white blood cell (WBC) count with mildly lower lymphocyte percentage count of 23.9%. Initial hemoglobin (Hb) was 10 mg/dL with a normocytic normochromic picture. Her hepatic and renal function tests as well as erythrocyte sedimentation rate (ESR) and serum adenosine deaminase (ADA) were within normal range. A spot and morning sputum test for acid-fast bacilli (AFB) and GeneXpert were negative with few Gram-positive cocci seen on the Gram stain. Chest X-ray showed nonhomogeneous cystic and nodular infiltrates at the left mid zone (Figure 1).

**Figure 2:**
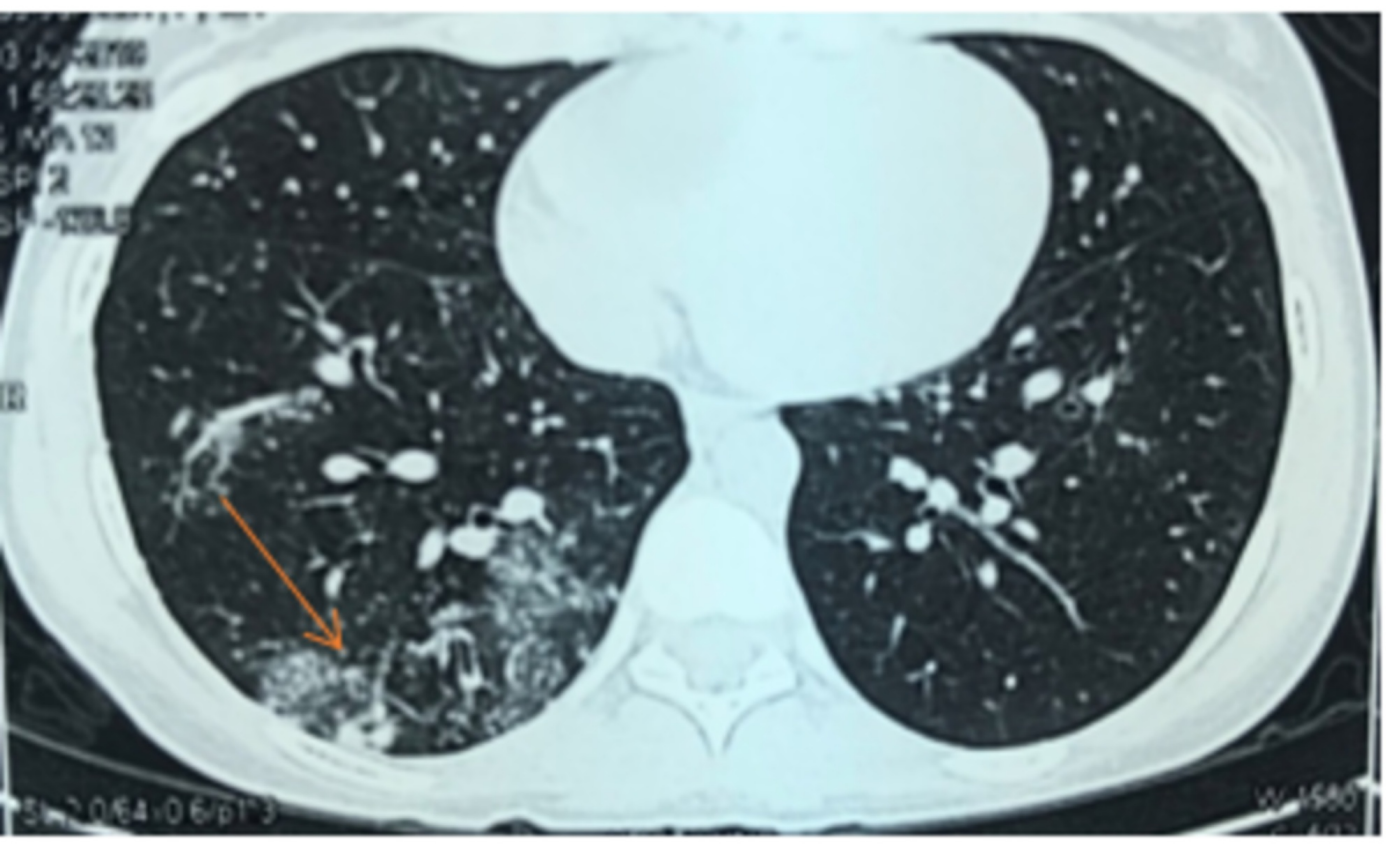
CT scan of the chest shows nodular infiltrates in the right lung with ground glass opacity.

**Figure 3:**
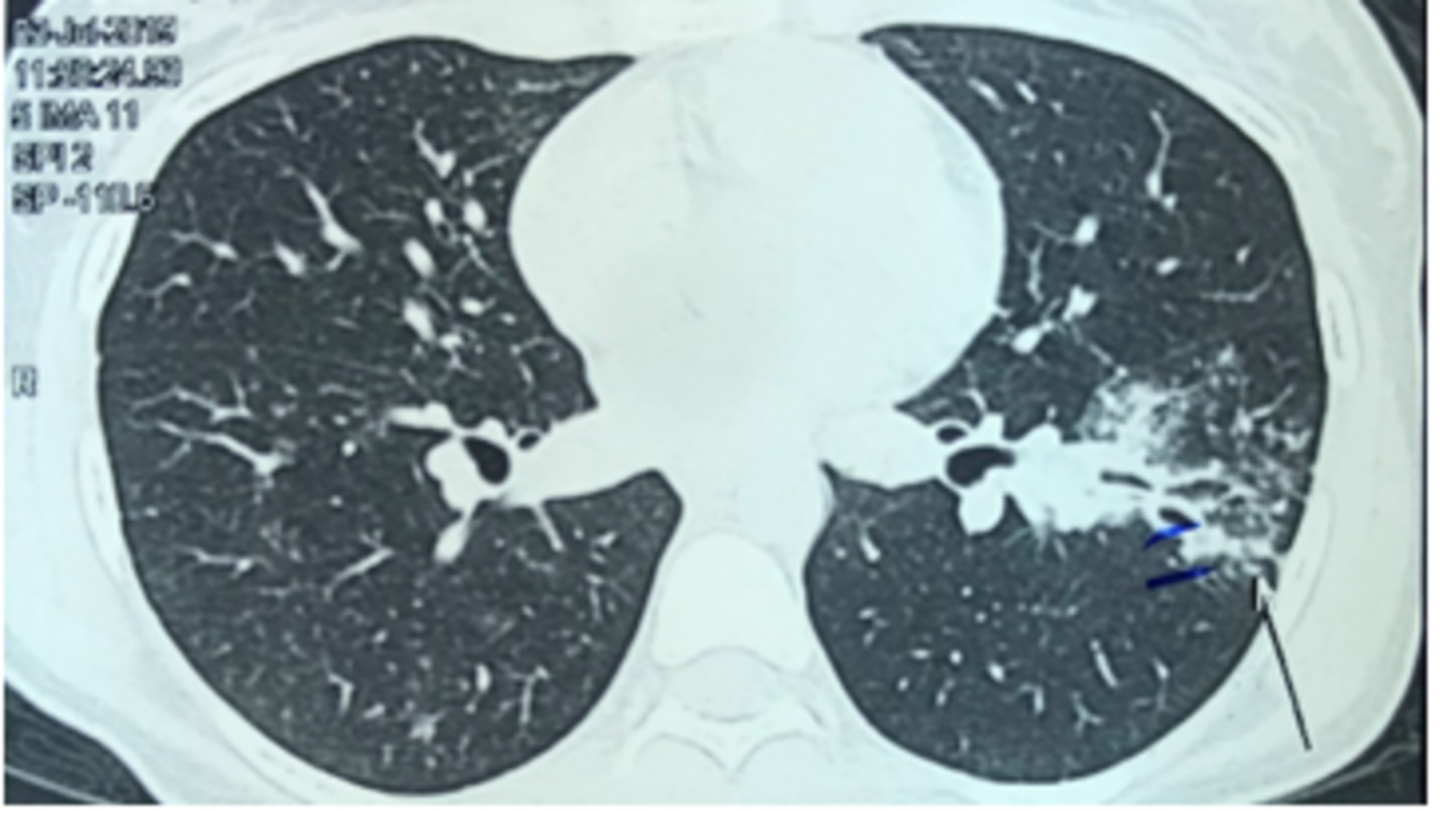
Patchy nodular infiltrates in the left lung along with traction bronchiectasis.

CT scan of the chest shows nodular infiltrates in the right lung with ground glass opacity (Figure 2) and patchy nodular infiltrates in the left lung along with traction bronchiectasis (Figure 3).

 

 

**Figure 4:**
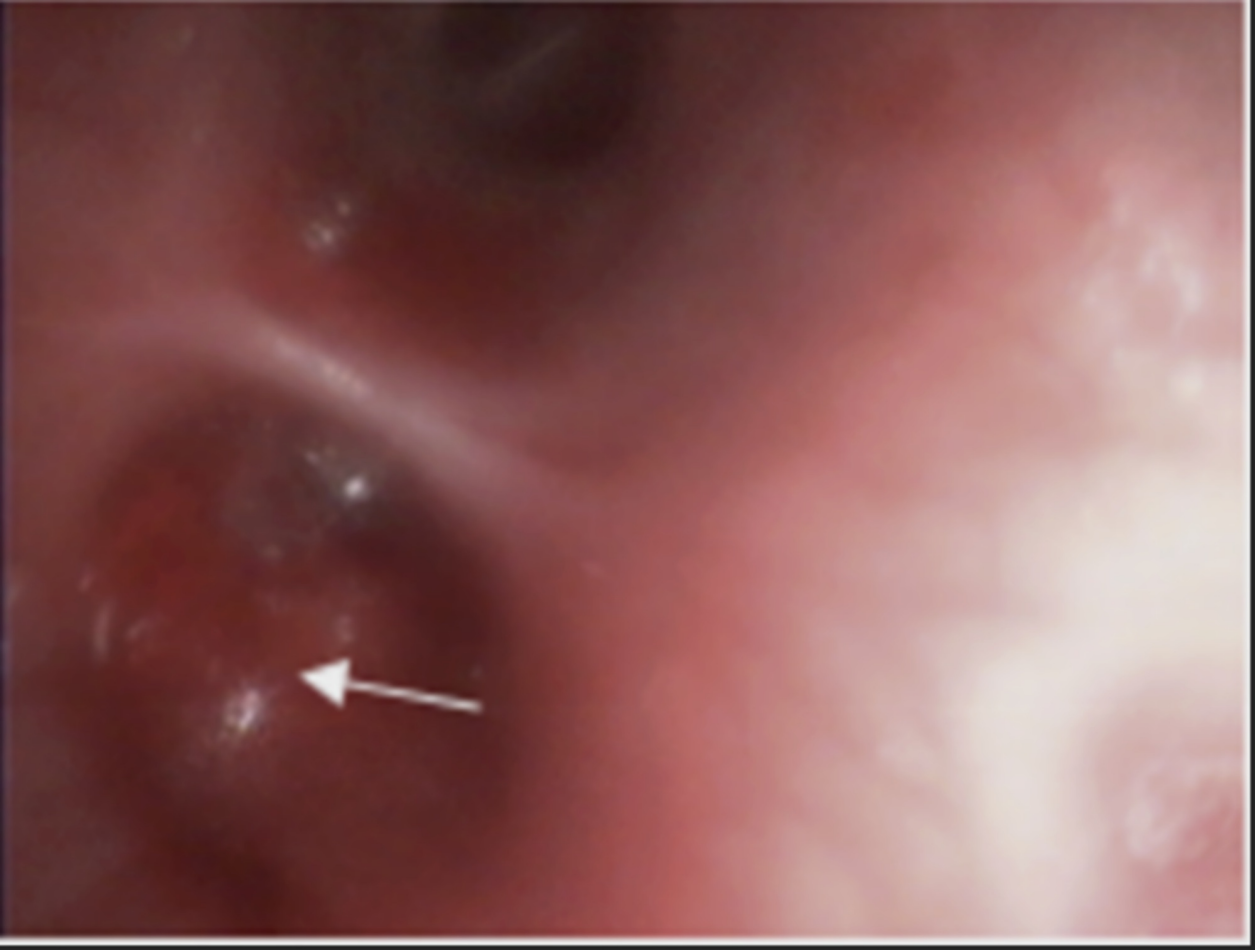
Endobronchial mass seen in the left lower lobe.

Eventually bronchoscopy was performed which showed significant bleeding in the presence of an endobronchial tumor in the left lower lobe (Figure 4). Biopsy did not reveal signs of malignancy or a granuloma while Ziehl-Neelsen staining was negative again for AFB. Bronchoalveolar lavage sample was positive for AFB and nucleic acid amplification using GeneXpert was low positive for *M. tuberculosis* and no rifampicin resistance was seen. Culture in Löwenstein-Jensen medium also confirmed the presence of *M. tuberculosis* complex.

While awaiting her work up she was empirically treated as a case of pneumonia with various antibiotics as an outpatient for which she did not show much favorable response. The patient was started on standard anti-tuberculosis treatment with isoniazid, rifampicin, ethambutol, and pyrazinamide as per national standard treatment guidelines. In a month’s time, the patient reported a steady improvement in her condition. She was subsequently discharged and closely followed up in the clinic where she reported significant improvement in her condition with no further episodes of hemoptysis. The patient denied respiratory distress and clinical features of bronchial stenosis during the course of treatment.

## Discussion

The reported incidence of EBTB is between 5.8% and 30% of all cases of *M. tuberculosis* [7]; most cases that have been reported are aged less than 35 years with children being at a greater risk of developing EBTB and with a higher female preponderance [8-9]. Among the elderly patients with TB, 15% of them were found to have EBTB [10]. EBTB diagnosis remains a challenge and is often underdiagnosed, as fiberoptic bronchoscopy is not typically performed in all patients with tuberculosis [11].

There is also limited data on the clinical manifestation of EBTB. The most common symptom is barking cough; yet, this is a nonspecific feature, and so are the chest pain, blood-streaked sputum, generalized weakness, dyspnoea, and fever; however, longer duration of symptoms in patients with pulmonary tuberculosis is the main predictor of concomitant EBTB [9]. Patients with endobronchial mass in some cases may have localized wheeze or rhonchi confusing it with bronchial asthma. In rare cases patients have coughed up fragments of bronchial cartilage [4, 12].

Chest radiograph may be normal in about 10%-20% of patients with EBTB [11].The most common abnormality on chest X-ray in EBTB is the presence of parenchymal infiltrates or consolidation in the affected lobe as was the case in our patient. Other findings may be present such as segmental or lobar collapse due to bronchial stenosis [4, 13].

In terms of radiological diagnosis, high resolution computed tomography (HRCT) has been found to be superior to chest radiographs and standard CT which not only assists in the localization of disease but also in the evaluation of parenchymal disease. Various studies done have reported a higher sensitivity of more than 95% in evaluation of EBTB by HRCT [14-15]. Therefore, an attempt should be made to rule out EBTB in all cases of endobronchial lesion. Bronchoscopy and biopsy are mandatory for the diagnosis of EBTB, though the yield of biopsies to diagnose EBTB ranges from 30% to 84% [2, 10]. Histopathological findings aid in the early diagnosis and prompt starting of treatment [16]; this was certainly not with our case where the diagnosis was confirmed by culture and nucleic acid amplification of broncho alveolar lavage. Pulmonary function test (PFT) shows restrictive lung disease pattern among individuals with EBTB. In our patient PFT was not performed [2, 17-18].

The common complications of EBTB are bronchial stenosis and stricture formation, fortunately this phenomenon did not develop in our patient. The stenosis can be fatal and may lead to respiratory compromise if larger airways are involved; of note postobstructive bronchiectasis can also manifest as a sequelae [2]. Chung and Lee have classified EBTB into seven different subtypes: actively caseating, edematous hyperemic, fibro stenotic, tumorous, granular, ulcerative, and nonspecific bronchitis. Formation of the granulation tissue is the main factor which classifies the subtypes, though any subtypes can be transform into another depending on disease progression or healing [11, 19].

The treatment of EBTB is the same as pulmonary tuberculosis [2, 18]. It involves a fixed dose regimen of four primary drugs which include: isoniazid, rifampicin, ethambutol, and pyrazinamide for an initial two months and then a continuation phase of treatment with daily isoniazid and rifampicin for a further four months. Beneficial effects of anti-inflammation with corticosteroids have been reported, however, were not used in our patient. In drug resistant cases, treatment should be based on susceptibility results.

The patient’s course of disease depends on the histopathological findings. The ulcerative pattern is the least encountered form, whereas active caseating is the most common subtype seen. Interventional bronchoscopy has been an emerging therapy and surgery is considered for refractory cases [2, 4].

## Conclusions

Endobronchial tuberculosis is encountered mainly among young females as is the case in our patient. It remains undiagnosed as the AFB smears are negative and diagnostic bronchoscopy is not routinely performed in those patients, mainly in the developing countries. Nevertheless, anti-tuberculosis therapy remains the first choice. Interventional procedure via bronchoscopic route with laser, argon, and stent placement to relieve scarring and stenosis remains an option and surgical procedure is reserved as the last resort for refractory cases. Clinicians need to be vigilant in patients who are AFB smear negative, with symptoms and localized wheeze; bronchoscopy should be considered in those selected cases.
